# Life course socioeconomic inequalities and oral health status in later life: ELSI-Brazil

**DOI:** 10.11606/S1518-8787.2018052000628

**Published:** 2018-10-25

**Authors:** Fabíola Bof de Andrade, José Leopoldo Ferreira Antunes, Paulo Roberto Borges de Souza, Maria Fernanda Lima-Costa, Cesar de Oliveira

**Affiliations:** I Fundação Oswaldo Cruz. Instituto René Rachou. Núcleo de Estudos em Saúde Pública e Envelhecimento. Belo Horizonte, MG, Brasil; II Fundação Oswaldo Cruz. Instituto René Rachou. Programa de Pós-Graduação em Saúde Coletiva. Belo Horizonte, MG, Brasil; III Universidade de São Paulo. Faculdade de Saúde Pública. Departamento de Epidemiologia. São Paulo, SP, Brasil; IV Fundação Oswaldo Cruz. Instituto de Comunicação e Informação Científica e Tecnológica em Saúde. Rio de Janeiro, RJ, Brasil; V University College London, Department of Epidemiology & Public Health. London, UK

**Keywords:** Aged. Tooth Loss, epidemiology. Mouth, Edentulous, epidemiology. Denture, Complete, utilization. Health Status Disparities

## Abstract

**OBJECTIVE:**

To investigate the association between life course socioeconomic conditions and two oral health outcomes (edentulism and use of dental prostheses among individuals with severe tooth loss) among older Brazilian adults.

**METHODS:**

This was a cross-sectional study with data from the Brazilian Longitudinal Study of Aging (ELSI-Brazil) which includes information on persons aged 50 years or older residing in 70 municipalities across the five great Brazilian regions. Regression models using life history information were used to investigate the relation between childhood (parental education) and adulthood (own education and wealth) socioeconomic circumstances and edentulism and use of dental prostheses. Slope index of inequality and relative index of inequality for edentulism and use of dental prostheses assessed socioeconomic inequalities in both outcomes.

**RESULTS:**

Approximately 28.8% of the individuals were edentulous and among those with severe tooth loss 80% used dental prostheses. Significant absolute and relative inequalities were found for edentulism and use of dental prostheses. The magnitude of edentulism was higher among individuals with lower levels of socioeconomic position during childhood, irrespective of their current socioeconomic position. Absolute and relative inequalities related to the use of dental prostheses were not related to childhood socioeconomic position.

**CONCLUSIONS:**

These findings substantiate the association between life course socioeconomic circumstances and oral health in older adulthood, although use of dental prostheses was not related to childhood socioeconomic position. The study also highlights the long-lasting relation between childhood socioeconomic inequalities and oral health through the life course.

## INTRODUCTION

Oral diseases are among the most common chronic diseases worldwide^1^. Healthy teeth are crucial for quality of life^2^, particularly for an adequate diet^3^ and functioning^4^. Poor oral health is a major global health burden^1^. Severe tooth loss is ranked in the 36th position among the 100 chronic diseases that affect life expectancy^1^. Impaired oral health is especially important among socioeconomic disadvantaged groups and follows a gradient in which lower socioeconomic groups have worse oral health^5^.

A person’s socioeconomic position (SEP) at different stages of life course has been found to be associated with general health^6^ and with an increased risk of tooth loss.^7^ Therefore, the life course approach has gained considerable attention in understanding social inequalities in oral conditions^8^.

The relation between early and later life SEP – which is measured through parental education, income, or own education – and oral health has been evaluated by few studies mostly conducted in high-income countries and using the oral health of adolescents and adults as outcomes^9–12^. Moreover, the magnitude of life course SEP inequalities on edentulism among older adults has not been well explored and most studies have used single life course SEP measures. Parental occupation has been reported to be associated with tooth loss in adulthood^13^. Both childhood and adult SEP are related to edentulism among adults^5^ and older individuals^14^. Recently, a study has assessed the magnitude of socioeconomic inequalities in edentulism and tooth loss using three life course measures representing different life stages (i.e., parental education, own education, and income). Relative inequalities were significant for parental education with a higher prevalence of edentulism among individuals whose parents had a lower level of education^7^.

The importance of early and later life SEP for the magnitude of inequalities needs to be explored for the appropriate planning and evaluation of public health interventions and for the reduction of the impact of SEP on oral health. Few studies have simultaneously assessed early, mid, and later life SEP in relation to edentulism^7^ ^,^ ^8^ and none of them has explored these measures in relation to the use of dental prostheses. Each SEP measure has different pathways in the association with oral health. Early life SEP may shape a child’s oral health as it directly impacts prevention practices^8^ ^,^ ^15^, dental care visit^15^, and, consequently, tooth loss in the permanent dentition at younger ages^13^. Therefore, the objective of this study was to investigate the association between life course socioeconomic circumstances and two oral health measures in later life, i.e., edentulism and use of dental prostheses among community-dwelling Brazilians aged 50 years or older.

## METHODS

### Study Population

This was a cross-sectional analysis with data from the baseline survey of the Brazilian Longitudinal Study of Aging (ELSI-Brazil). This is a nationally representative, population-based cohort study of persons aged 50 years or older. The baseline survey was conducted between 2015 and 2016. The sample comprised 9,412 individuals residing in 70 municipalities from different Brazilian regions. The sampling procedure combined geographical stratification and clustering. The primary sampling units were municipalities, and census tracts were the second stage followed by households. All residents in the selected households aged 50 years or older were eligible for an interview and other procedures. Detailed information regarding the ELSI-Brazil can be found elsewhere^16^ ^,^ ^a^. All interviews were conducted at the participant’s homes by trained interviewers. The study questionnaire included the following information: sociodemographic and behavioral characteristics, lifestyle, quality of life, use of health services, and general health information.

The analytical sample included all participants aged 50 years or older with complete information for analysis related to the two outcomes of interest. The final sample for edentulism was n = 8,399 and for use of dental prostheses among individuals with severe tooth loss was n = 6,114. Regarding the analysis for edentulism, there was a significant difference between those included and excluded because of missing data with regard to age and own education. Individuals in the group of 70 years or more had fewer chances to be included when compared to those in the group of 50 to 59 years. Those with 8–11 and 12 or more years of schooling had higher chances to be included when compared to those with 0–3 years of schooling. The same differences were found for use of dental prostheses, but the significance was found only for the 12 or more years of schooling.

### Oral Health Measures

Oral health measures were collected via self-reported questions on the number of teeth and use of dental prostheses. Edentulism was defined as no teeth in the mouth. Edentulous individuals were asked about the time since edentulism with the following possible answers: 1) ≤ 6 months, 2) seven months – one year, 3) 2–5 years, 4) 6–9 years, and 5) 10 or more years.

Use of dental prostheses was evaluated among individuals with severe tooth loss defined as the presence of fewer than 20 teeth^17^. The presence of 20 teeth is considered the minimum number of natural teeth that are needed to maintain function without prosthetic appliances.

All individuals with less than 20 teeth were asked about the use of removable dental prostheses. Individuals reporting the use of dental prostheses were asked to report the place of rehabilitation which was categorized as either public or private service.

### Life Course Socioeconomic Position Measures

Life course socioeconomic status was assessed in terms of three indicators: parental education, own education, and wealth. Parental education was considered a proxy measurement of socioeconomic circumstances during childhood^7^. This indicator has reported to be the most commonly used variable to assess socioeconomic position during childhood^18^. This characteristic was classified according to the participant’s response to the following question: “What is (was) the level of education of you father (mother)?”. Six alternative responses were offered: 1) no formal education, 2) incomplete primary school, 3) complete primary school, 4) complete secondary school, 5) complete tertiary school, and 6) university degree or higher. Options 4, 5, and 6 were categorized as complete secondary school or more because of the lower frequencies of these options.

The participant’s own level of education was used to assess socioeconomic position during adolescence and early adult life. Most epidemiological studies have adopted this option, even though some individuals go to college or complete earlier stages during late adulthood^18^. It was based on self-reported number of years of schooling years or categorized as follows: 0–3 years, 4–7 years, and 8 or more years. Wealth was used as an indicator of later life socioeconomic position. Previous research has considered this measure to be more sensitive than income in predicting socioeconomic inequality for health-related outcomes^19^. The wealth status index was constructed via principal components analysis^20^ using information on household ownership of durable goods and housing characteristics based on the following information: household assets [Internet, television, cable television, refrigerator, washing machine, dishwasher, tumble dryer, computer, desk phone, cell phone, microwave, motorcycle, car] and household characteristics [presence of a maid, masonry wall or wood wall, piped water, paved street, bathroom]. The wealth variable was categorized into quintiles.

### Statistical Analysis

Statistical analyses involved descriptive and inferential analyses with a 5% significance level and 95% confidence interval (95%CI). Descriptive statistics were estimated for both outcomes and covariates (age and sex). The description of the self-reported time since edentulism and the place of dental rehabilitation were also presented. Associations between categorical variables were assessed using the Rao-Scott chi-square test. Adjusted prevalence of edentulism and use of dental prostheses according to socioeconomic rank were also reported.

The absolute and relative magnitudes of socioeconomic inequality in oral health were measured using the slope index of inequality (SII) and the relative index of inequality (RII), respectively. Each category of socioeconomic measure is assigned a ridit score based on the midpoint of the range in the cumulative distribution of the population in the given category. Individuals were cumulatively ranked from 0 to 1 according to ascending socioeconomic position such that “0” represented the lowest level and “1” represented the highest. The ridit score was then entered as an independent variable in the regression model. The SII is the difference in the prevalence of functional dentition (absolute inequality) and RII is the prevalence ratio (relative inequality) between those at the top rank (one) and those at the lowest rank (zero). A SII value greater than zero and a RII value greater than one indicate that the prevalence of the outcome is greater in the group with higher socioeconomic status.

The SII and RII were estimated for each oral health outcome using Poisson regression models^21^. The model was adjusted for age and sex taking into consideration their relation with both oral health outcomes and socioeconomic position. First, we estimated the association between outcomes and each SEP individually adjusted for age and sex. These models are identified as Model 1 in the tables and figures. Then, we estimated one final model, for each outcome, simultaneously adjusted for all SEP. Only the significant SEP were kept in the final models (Model 2). All statistical analyses were performed using Stata, version 13.0 (StataCorp LP, College Station, Texas, USA) using the *svy* command to analyze data originating from a complex sample.

### Ethical Issues

The ELSI-Brazil was approved by the ethics board of Oswaldo Cruz Foundation, Minas Gerais (CAAE 34649814.3.0000.5091). Genotyping of the cohort population was approved by the Brazilian national research ethics committee (CAAE 63725117.9.0000.5091). Participants signed separate informed consent forms for the interviews, physical measurements, and laboratory assays, and they authorized sample storages and access to administrative records.

## RESULTS

Table 1 and Table 2 describe the distribution of the population and the dependent variables according to demographic characteristics and socioeconomic status. For both samples, most of the individuals were female and had less than eight years of schooling. Approximately half of the sample reported that their parents did not have formal education.

**Table 1:** Description of the sample and distribution of edentulism according to independent variables. Brazilian Longitudinal Study of Aging (ELSI-Brazil), 2015–2016. (n = 8,399)

Characteristics	Total	Edentulism
	
	% (95%CI)	% (95%CI)
Age (years)		
50–59	48.3 (44.1–52.6)	15.5 (13.6–17.6)*
60–69	29.7 (27.8–31.7)	32.9 (29.9–36.0)
≥ 70	22.0 (19.4–24.9)	53.2 (49.9–56.5)
Sex		
Male	46.2 (43.1–49.3)	21.6 (19.1–24.4)*
Female	53.8 (50.7–56.9)	35.2 (32.5–38.0)
Own education (years)		
0–3	32.0 (28.5–35.6)	45.3 (41.8–48.8)*
4–7	31.1 (28.6–33.8)	31.6 (28.8–34.4)
8–11	28.1 (25.5–30.9)	14.3 (12.4–16.5)
≥ 12	8.7 (7.6–10.1)	6.9 (5.1–9.3)
Parental education		
No formal education	49.4 (46.0–52.8)	36.3 (33.3–39.3)*
Incomplete primary school	22.4 (20.4–24.6)	26.7 (23.6–30.2)
Complete primary school	19.4 (17.3–21.7)	21.7 (18.8–25.0)
Complete secondary school or more	8.8 (7.7–10.1)	9.2 (6.7–12.5)
Wealth		
1st quintile (poorest)	19.4 (15.3–24.2)	41.4 (37.0–45.9)*
2nd quintile	20.0 (18.0–22.1)	38.2 (34.4–42.1)
3rd quintile	19.8 (18.0–21.7)	30.6 (27.3–34.1)
4th quintile	20.4 (18.0–23.0)	21.6 (18.8–24.8)
5th quintile (richest)	20.5 (17.7–23.6)	13.9 (11.5–16.7)

* p < 0.001

**Table 2:** Description of the sample and distribution of use of dental prostheses among individuals with severe tooth loss, according to independent variables. Brazilian Longitudinal Study of Aging (ELSI-Brazil), 2015–2016. (n = 6,114)

Characteristics	Total	Use of dental prostheses
	
	% (95%CI)	% (95%CI)
Age (years)		
50–59	40.0 (36.2–43.8)	81.6 (78.3–84.5)
60–69	32.5 (30.8–34.3)	82.6 (78.8–85.8)
≥ 70	27.5 (24.7–30.5)	79.7 (75.9–83.0)
Sex		
Male	42.2 (39.6–44.8)	73.9 (70.1–77.5)*
Female	57.8 (55.2–60.4)	86.8 (84.0–89.2)
Own education (years)		
0–3	40.1 (36.4–43.9)	72.8 (68.8–76.5)*
4–7	33.8 (30.7–37.1)	85.3 (81.6–88.4)
8–11	21.8 (19.5–24.3)	89.7 (87.5–91.5)
≥ 12	4.3 (3.6–5.1)	88.1 (82.8–91.9)
Parental education		
No formal education	55.4 (51.6–59.2)	77.9 (74.2–81.2)*
Incomplete primary school	22.8 (20.7–25.0)	83.6 (80.6–86.3)
Complete primary school	16.7 (14.3–19.4)	88.4 (84.6–91.3)
Complete secondary school or more	5.1 (4.3–6.0)	86.9 (80.9–91.2)
Wealth		
1st quintile (poorest)	23.7 (18.9–29.2)	66.0 (61.1–70.5)*
2nd quintile	23.2 (21.1–25.5)	80.6 (76.9–83.8)
3rd quintile	20.6 (18.3–23.0)	86.5 (83.2–89.3)
4th quintile	18.8 (16.5–21.4)	89.6 (87.0–91.7)
5th quintile (richest)	13.7 (11.6–16.2)	90.5 (87.8–92.6)

* p < 0.001

**Table 3:** Relative inequalities (RII) related to edentulism and use of dental prostheses among individuals without functional dentition. Brazilian Longitudinal Study of Aging (ELSI-Brazil), 2015–2016.

Variable	Edentulism	Use of dental prostheses
	
	RII (95%CI)	RII (95%CI)
Model 1^a^		
Parental education	0.40 (0.32–0.49)	1.24 (1.14–1.35)
Own education	0.25 (0.21–0.29)	1.39 (1.28–1.51)
Wealth	0.34 (0.28–0.42)	1.48 (1.35–1.61)
Model 2^b^		
Parental education	0.77 (0.64–0.93)	-
Own education	0.37 (0.32–0.43)	1.20 (1.11–1.28)
Wealth	0.57 (0.46–0.70)	2.37 (1.27–1.49)

^a^ Model 1: individual models for the association of each socioeconomic position indicator with oral health. Models were adjusted for age and sex. The model for use of dental prostheses was also adjusted for the number of teeth.

^b^ Model 2: final model for the association of oral health and socioeconomic position indicators. Models were mutually adjusted for all socioeconomic position indicators. Models were adjusted for age and sex. The model for use of dental prostheses was also adjusted for the number of teeth.

In relation to oral health conditions, 28.8% (95%CI 26.5–31.5) were edentulous and among those with severe tooth loss 80% (95%CI 78.5–84.0) used dental prostheses. Most dental rehabilitation (91.7%) was done in the private sector, 7.7% reported the private service, and 0.6% other services. Regarding the time since edentulism, 76.9% reported 10 years or more, followed by individuals reporting two to five years, six to nine years (6.6%), seven months to one year (2.4%), and ≤ 6 months (1.8%).

**Figure 1:**
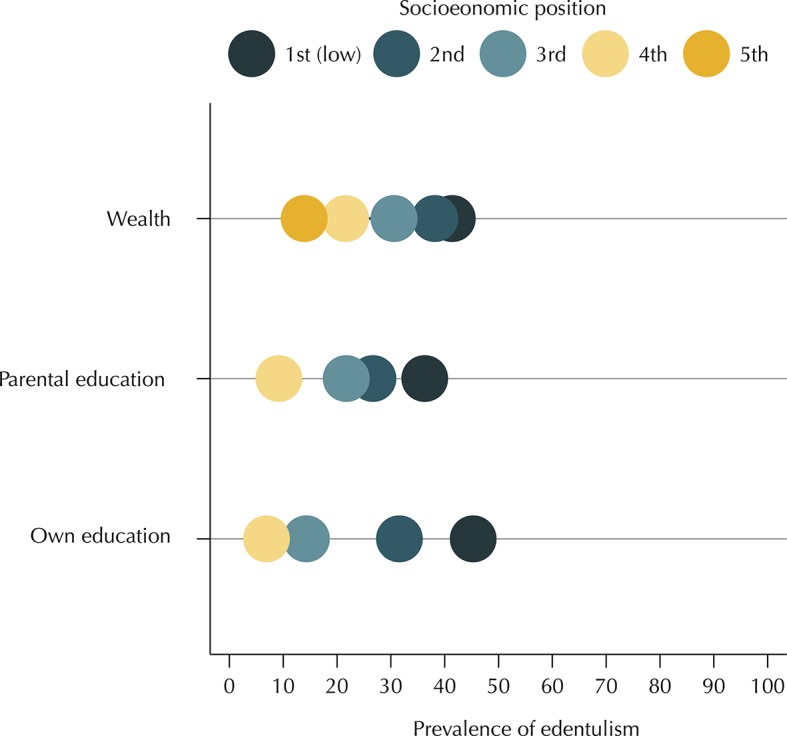
Prevalence of edentulism according to socioeconomic position (equiplot). Brazilian Longitudinal Study of Aging (ELSI-Brazil), 2015–2016.

**Figure 2:**
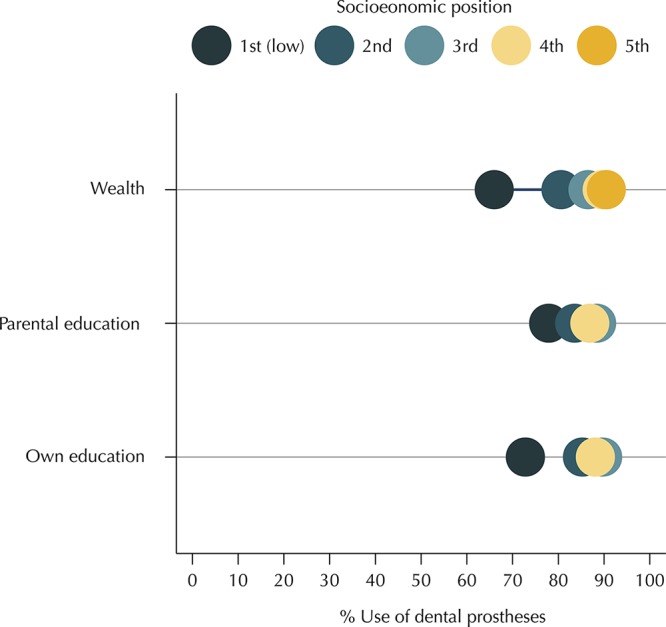
Prevalence of use of dental prostheses among individuals with severe tooth loss according to socioeconomic position (SEP) (equiplot). Brazilian Longitudinal Study of Aging (ELSI-Brazil), 2015–2016.

Figures 1 and 2 show, respectively, the prevalence of edentulism and use of dental prostheses according to the levels of SEP. Prevalence of edentulism decreased with an increase in the level of SEP for all measures. An inverse relation was found between SEP indicators and the use of dental prostheses.

**Figure 3:**
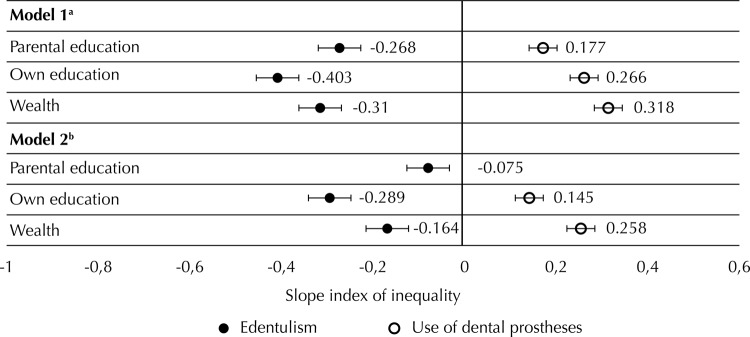
Absolute inequalities (SII) related to edentulism and use of dental prostheses among individuals with severe tooth loss. Brazilian Longitudinal Study of Aging (ELSI-Brazil), 2015–2016.

^a^ Model 1: individual models for the association of each socioeconomic position indicator with oral health. Models were adjusted for age and sex. The model for use of dental prostheses was also adjusted for the number of teeth.

^b^ Model 2: final model for the association of oral health and socioeconomic position indicators. Models were mutually adjusted for all socioeconomic position indicators. Models were adjusted for age and sex. The model for use of dental prostheses was also adjusted for the number of teeth.

Figure 3 shows the absolute inequalities (SII) related to both oral health outcomes. In Model 1, in which the socioeconomic position measures were analyzed separately, differences in prevalence were significant for all three measures, and the highest difference was found for own education in relation to edentulism and wealth regarding the use of dental prostheses. The prevalence of use of dental prostheses was approximately 32 percent points higher (SII = 0.318, 95%CI 0.254–0.382) in the wealthiest individuals compared to those at the bottom level. In Model 2, the three socioeconomic measures were included simultaneously leading to an attenuation of the absolute inequalities with a reduction in the differences in prevalence. The attenuation was the highest for parental education, which also became non-significant in relation to the use of dental prostheses. Similar patterns were observed for relative inequalities (RII) (Table 2).

## DISCUSSION

In this large population-based cohort of Brazilian older adults aged 50 years or older, we observed the existence of significant inequalities in edentulism and use of dental prostheses among individuals with severe tooth loss using different life course socioeconomic measures. The findings highlight that the magnitude of edentulism is higher among individuals with lower levels of SEP during childhood, irrespective of their current SEP. These findings are the most relevant results of this study. It is relevant to acknowledge the enduring impact of SEP inequalities during the life course on oral health of older adults for health policies and planning, in special in the targeting of resources to population groups with higher levels of dental treatment needs.

The results reported here concur with reports from other countries, which also depicted socioeconomic inequalities in both edentulism and use of dental prostheses among individuals with severe tooth loss^5^ ^,^ ^14^. A previous study in Korea^7^ has assessed absolute and relative inequalities in edentulism and severe tooth loss when mutually adjusted for SEP indicators related to different stages of the life course. Although observing an overall reduction in the magnitude of the inequalities, both the SII and the RII remained statistically significant for severe tooth loss, whereas absolute inequalities (SII) were not significant for edentulism. However, the lack of significant absolute SEP inequalities for edentulism in the Korean population may be related to the low prevalence of this outcome in the country, because the SII depends on the overall level of the outcome, and the RII for edentulism remained statistically significant even after mutually adjusting for the SEP measures related to different stages of the life course. Our findings corroborate previous findings that have demonstrated that both own education and parental SEP are related to edentulism^5^, although inequalities related to the former were stronger. The findings support the hypotheses that the impact of childhood socioeconomic circumstances on the oral health of adults may be small, but they have long-lasting negative influences^8^ ^,^ ^22^.

The attenuation in the magnitude of early life SEP inequalities when adjusted for own education and wealth may be explained by the cumulative pattern of oral health impairments and the ability of each SEP measure to keep influencing the changing oral health patterns and behavioural factors. However, as edentulism occurred at adulthood for most Brazilian adults in the study and it is the final stage of oral disease and dental treatment, it is plausible that own education and wealth have higher effects on edentulism as they are more proximal to the outcome. Own education may reflect parental education^23^, which demonstrates a life course continuity in SEP. In line with that, Poulton et al.^8^ have found that upward SEP mobility did not mitigate or reverse the adverse effects of low childhood SES on adult oral health.

Education can also act through different pathways, such as improving health-related knowledge in adult life and allowing for favorable employment opportunities with higher income levels and better living conditions throughout adult life^24^, which can impact tooth loss. These different pathways may explain the higher difference in the prevalence of edentulism in relation to own education when compared to wealth as both measures may share similar pathways, but the former has a long-lasting affect and may also determine wealth. The concurrent significance of wealth-related inequalities in edentulism may be explained by the fact that education is not only a measure of material affordability and sometimes it is not a good measure of it as this concept has different social meaning across time and cultures^23^. Wealth at the age of 50 years or older may reflect the final level of SEP of the population and it is more related to the individual’s material circumstances in old age because of the impact of retirement on income^25^. Accordingly, the wealthiest individuals have more material resources for health-enhancing commodities and access to health care^26^.

Regarding the use of dental prostheses, there was no association with parental education when the model was adjusted for all SEP measures and the magnitude of inequality was higher for wealth than for own education. These results and the one showing that most prosthetic replacements were made in the private sector support the hypotheses that the demand for dental treatment is mostly driven by the ability of the patient to afford the costs. A previous study has found that the level of inequality related to wealth for dental care is higher than that related to income as it is more related to the ability to create disposable income^25^. Nonetheless, the association with own education suggests that inequality in the use of dental prostheses cannot be explained by economic budget constrain alone, and some part might be related to social and behavioral norms^27^. Brazil has a Universal Health Coverage System and, since the release of the National Oral Health Policy in 2004, removable prosthetic replacement is available free of charge in public services. Although there have been improvements, as 2.1 million dental prostheses were delivered to adult and older individuals up to 2015^28^, our findings highlight that this service still has a small impact on the population needs, as approximately 92% of the prostheses were made in private services.

Our study has several strengths and potential limitations that need to be considered. A major strength is the large and representative sample of community-dwelling Brazilian men and women aged 50 years or older using a wide range of covariates. To the best of our knowledge, this study is the first in Latin America to investigate socioeconomic inequalities in oral health status later in life using different life course SEP measures. In addition, certified interviewers following standardized protocols, thus assuring excellent quality of data, performed all measurements. Limitations could be attributed to the fact that data collected on childhood circumstances and oral health may be subject to information bias. In particular, we relied on recalled data on parental education for the early childhood socioeconomic position. Thus, the impact of childhood socioeconomic position on tooth loss might have been underestimated. Regarding the oral health data, the self-report of the number of teeth has been described as strongly correlated with clinical records on the exact number of missing teeth^29^.

In summary, the findings of this study showed an association between life course socioeconomic inequalities for both tooth loss and use of dental prostheses in a nationally representative sample of Brazilian older adults. Although use of dental prostheses was not related to childhood SEP, it was significantly associated with two other life course SEP, own education and wealth, which were used to represent socioeconomic position during adolescence or early adult life and later life, respectively. The study also highlights the importance of using different SEP measures, as they can express different dimensions of socioeconomic inequalities depending on the oral health outcome.

## Footnotes

a Fundação Oswaldo Cruz. Brazilian Longitudinal Study of Aging. Rio de Janeiro; c2015 [cited 2017 Nov 28]. Available from: http://elsi.cpqrr.fiocruz.br
